# The molecular similarity landscape of preclinical cancer models to patient tumors

**DOI:** 10.1172/jci.insight.206449

**Published:** 2026-06-30

**Authors:** Zixuan Xie, Jia Xue, Binchen Mao, Hengyuan Liu, Wubin Qian, Jingjing Wang, Xiaobo Chen, Sheng Guo

**Affiliations:** 1 Crown Bioscience Inc., Suzhou, Jiangsu, China.; 2 Biomedical Basic Research Center (BBRC) of Jiangsu, Suzhou, Jiangsu, China.

**Keywords:** Genetics, Oncology, Bioinformatics, Cancer, Mouse models

## Abstract

Selecting appropriate preclinical models is fundamental for translational oncology, yet a large-scale, multi-omic quantitative comparison of their similarity to primary human tumors is lacking. To address this, we integrated transcriptomic, proteomic, and genomic profiles from over 10,000 primary tumors from The Cancer Genome Atlas (TCGA) and the Clinical Proteomic Tumor Analysis Consortium (CPTAC), alongside 4,000 preclinical models. Using a robust computational framework, we revealed a clear hierarchy of transcriptomic and proteomic similarity to patient tumors: with patient-dervied xenografts (PDXs) having greater transcriptomic and proteomic similarity to patient tumors (>) compared with patient-derived organoids (PDOs), which are equal in hierarchy to that of PDX-dervied organoids (PDXOs) > cell lines. We also quantified high molecular conservation (Pearson correlation coefficient = 0.96) across paired in vitro to in vivo platform (organoids to PDX) transitions. Furthermore, genomic analysis demonstrated that whole-exome sequencing (WES) outperforms RNA-seq in detecting DNA variants, and it identified a clonal complexity hierarchy (cell lines > PDXOs > PDXs > PDOs) reflecting the effect of passaging history on intratumor heterogeneity. Ultimately, this study delivers a comprehensive quantitative benchmark, establishing a population-level hierarchy of molecular similarity between preclinical models and primary tumors and providing a data-driven reference for model selection. These findings offer a data-driven framework for selecting models that balance biological representativeness with experimental practicality.

## Abstract 2

This study compares common cancer research models and shows which most closely resemble patient tumors, helping scientists choose better tools for testing treatments.<br>

## Introduction

The success of translational oncology relies on the molecular representativeness of the preclinical models utilized during its discovery and development. The high failure rate of oncology drugs in clinical trials, where the vast majority fail during phase II/III clinical trial, highlights a gap between experimental systems and the patients they are meant to represent (1–4). The causes of this high failure rate are multifactorial and extend beyond the properties of preclinical models alone. However, a quantitative understanding of how different model platforms correspond molecularly to patient tumors remains an important knowledge gap.

Historically, the cornerstone of cancer research was the immortalized 2-dimensional (2D) cell line. These models offered advantages in terms of cost, scalability, and ease of genetic manipulation, providing a high-throughput solution to evaluate the therapeutic efficacy of candidate anticancer agents. Cell line–based platforms became the standard for predicting clinical responses to early targeted therapies and chemotherapeutics (5, 6). However, as the field transitioned into the era of precision medicine, the profound biological shortcomings of 2D cultures became a substantial liability (7). Without a 3D architecture and tumor microenvironment, cell lines often undergo culture-induced evolution, drifting toward a generic proliferative phenotype and losing the specific markers of the original disease (8). Furthermore, cell lines are typically established only from the more aggressive tumors and, therefore, fail to represent the complex tumor heterogeneity observed in the clinic (9). For these reasons, the establishment of cell lines is often not an appropriate strategy for personalized medicine applications, and their clinical predictive value has historically been limited (9).

To circumvent these issues and improve the drug development process, there has been a renewed interest in the patient-derived xenografts (PDXs). While PDX models are not new — studies conducted in the 1980s already demonstrated a high degree of correlation between the clinical response to drugs in patients and the response to the same drugs in their corresponding PDX models — in the recent 2 decades, they have become a preferred preclinical tool in translational research (9). By engrafting patient tumor fragments directly into immunodeficient mice, PDXs maintain the histological structure, stromal components, and clonal architecture of the original tumor (10). The systematic generation of PDX models has improved the study of inter- and intratumoral heterogeneity (11, 12). Additionally, large-scale screening utilizing the “Mouse Clinical Trial” (MCT) concept has demonstrated that PDX populations can accurately predict clinical trial drug responses (13). Integrating next-generation sequencing with these in vivo models also provides a strong framework for personalized cancer treatment (14, 15). However, PDXs are constrained by high costs, low throughput, and slow engraftment times (16).

Alongside the use of xenografts, the continuous evolution of in vitro technologies provided another solution through organoids. The development of this technology builds upon decades of foundational research. Early efforts in 3D culture demonstrated that growing epithelial cells in extracellular matrices was necessary to restore physiological tissue function in vitro (17, 18). The field subsequently advanced when researchers utilized pluripotent stem cells to generate self-organizing organ models (19, 20). A critical milestone for oncology occurred when it was discovered that single adult stem cells could self-organize into complex, organ-like structures in a dish (21). Building on these biological principles, patient-derived organoids (PDOs) emerged as a platform that combines the scalability of cell lines with the biological relevance of tumors (22). By closely mimicking the architectural and genetic features of the primary tumor, organoids bridge the gap between high-throughput in vitro systems and complex in vivo models (23, 24). Studies have demonstrated the utility of this technology in precision medicine, showing that PDOs can accurately predict patient clinical responses to targeted treatments with high sensitivity (24, 25). However, while organoids maintain genetic stability better than standard 2D cell lines, they often lack the complete immune and stromal microenvironments found in a murine host (26).

Today, researchers frequently utilize these platforms in a complementary fashion — leveraging the scalability of organoids for exploratory screening while employing matched PDXs for in vivo validation. However, this methodological flexibility has outpaced a comprehensive biological characterization of how these platforms quantitatively relate to one another. While the unique strengths and inherent biases of cell lines, PDXs, and organoids are heavily documented individually, the field currently lacks a systematic, pancancer roadmap that defines their hierarchy of similarity across multiple molecular layers. Researchers often transition between platforms without a precise understanding of the transcriptomic and proteomic shifts that may occur during these interconversions. In this study, we address this gap by conducting an extensive comparative multi-omic analysis. In this study, we performed a comprehensive pancancer and panmodel comparison using an extensive multi-omic dataset encompassing over 10,000 primary tumors from The Cancer Genome Atlas (TCGA) (27) and the Clinical Proteomic Tumor Analysis Consortium (CPTAC) (28), along with 4 thousand preclinical models including PDXs, PDX-derived organoids (PDXOs), PDOs, and cell lines. By integrating transcriptomic, proteomic, and genomic profiles, we identified the following order of molecular similarity: PDXs > PDOs = PDXOs > cell lines, where > refers to more transcriptomic and proteomic similarity to patient tumors. We further quantified the rate of “transcriptomic drift” over sequential passages and evaluated the robustness of functional pathway activities using single-sample gene set enrichment analysis (ssGSEA) (29). Our results demonstrate that, while minor drift occurs over time, the major transcriptomic and proteomic signatures of patient tumors are broadly maintained at the population level across patient-derived platforms. It is important to note that this study characterizes cohort-level molecular similarity rather than per-patient model fidelity; systematic paired patient-to-model comparisons remain an important direction for future work.

## Results

### Panmodel and Pancancer comparison of gene expression profiles.

**Figure 1:**
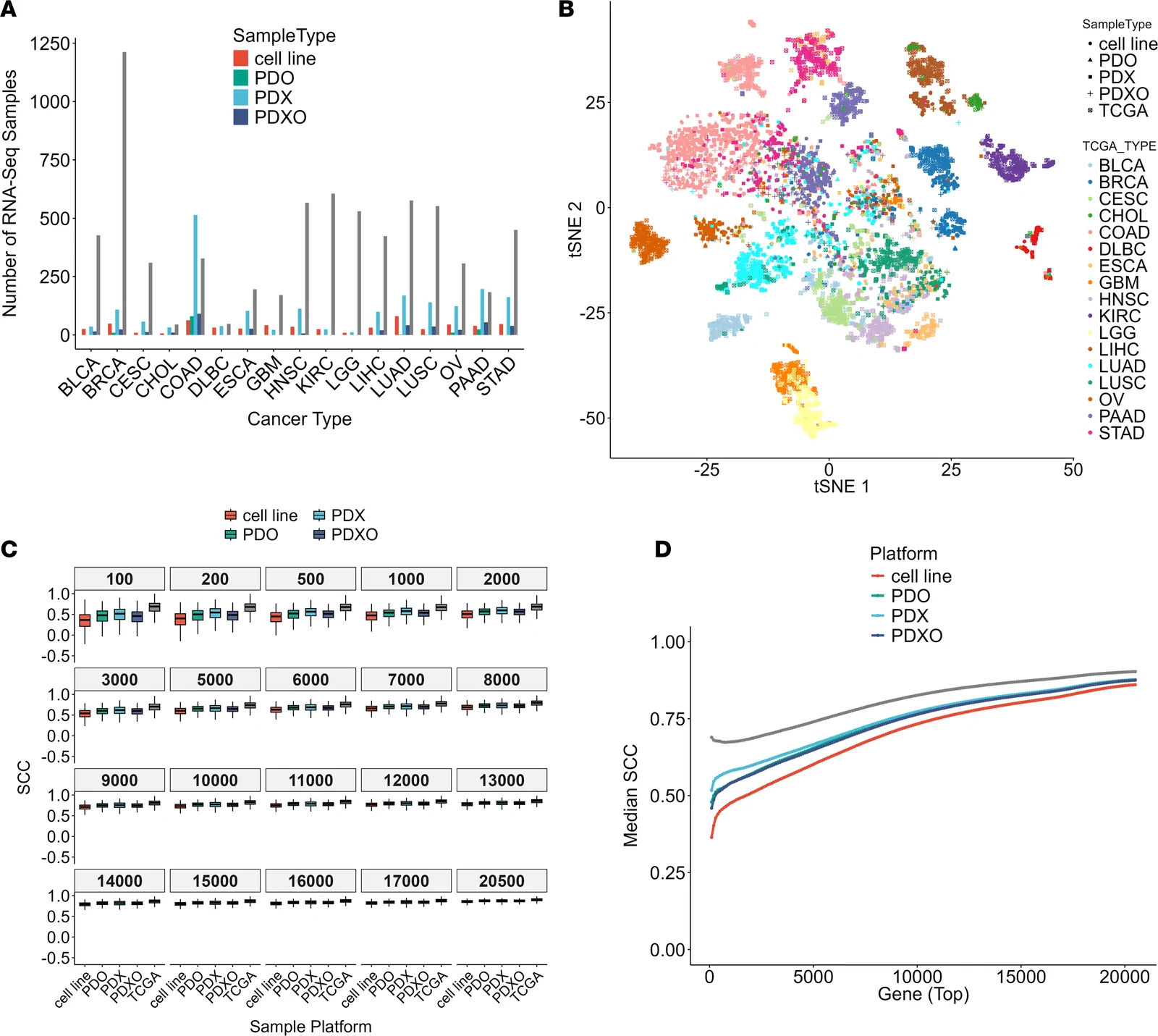
Pancancer analysis of different preclinical models and tumor samples. (**A**) Number of RNA-seq samples used, grouped by different model types and cancer types. (**B**) t-SNE plot for all RNA-seq samples across different TCGA cancer types. (**C**) Box plot of Spearman correlation coefficient between RNA-seq samples of TCGA and preclinical models for each model type, each panel represents the corresponding top *n* varied genes of TCGA samples. (**D**) Plot of median spearman correlation coefficient between TCGA and preclinical models against the number of top varied genes of TCGA samples.

We compared RNA-seq profiles from 10,626 primary tumors from TCGA with 2,762 PDX samples, 473 PDXO samples, 174 PDO samples from Crown Bioscience’s collection, and 1,170 cell lines from CCLE or Crown Bioscience’s collection, across 17 overlapping tumor types (Figure 1A and Table 1). The expression files were normalized using the Trimmed Mean of M values (TMM) method (30). The batch effects related to different model types were corrected using ComBat (31); an evaluation of the uncorrected RNA-seq data revealed that samples clustered entirely by data source rather than by biological tissue type (Supplemental Figure 1A; supplemental material available online with this article; https://doi.org/10.1172/jci.insight.206449DS1). To test whether ComBat correction removed biologically meaningful variance, we computed pairwise inter-cancer-type distances between TCGA centroids before and after correction for all 17 cancer types. The distances were virtually identical before and after correction (Pearson r = 0.996; Supplemental Figure 1B), demonstrating that the relative biological separation between cancer types was well preserved. To confirm that the correction did selectively remove platform-associated technical variance, we computed silhouette scores quantifying how strongly each sample clustered with its own platform relative to other platforms. Before correction, the median silhouette score was 0.18, indicating the platform separation. After correction, this dropped to approximately 0, confirming that platform-driven clustering was effectively eliminated (Supplemental Figure 1C). Taken together, these results demonstrate that ComBat correction in our study selectively removed technical batch effects while preserving the biologically meaningful inter-cancer-type variation that underlies all downstream comparisons.

To have an overview of the global transcriptional relationships among patient tumors and corresponding preclinical models, we performed the t-SNE analysis using the normalized and batch corrected gene expression profiles (Figure 1B). For most cancer types, samples clustered primarily according to their tumor type, indicating that, in general, the preclinical cancer models well represent the transcriptomic profiles of primary tumors. Major cancer types such as breast invasive carcinoma (BRCA), lung adenocarcinoma (LUAD), colon adenocarcinoma (COAD) and ovarian serous cystadenocarcinoma (OV) formed well-defined, compact clusters; in contrast, certain tumor types (e.g., cholangiocarcinoma (CHOL), lymphoid neoplasm diffuse large B-cell lymphoma (DLBC), cervical squamous cell carcinoma and endocervical adenocarcinoma (CESC) and esophageal carcinoma (ESCA) appeared relatively sparse or dispersed in the t-SNE space. Tumor type–specific t-SNE panels for all 17 cancer types are provided in Supplemental Figure 2, which also identifies outlier models within individual tumor types.

Though for each cancer type, the 4 preclinical models were generally positioned close to their corresponding TCGA tumors, the extent of similarity varied. To explore the order of transcriptomic similarity, we then calculated Spearman correlation coefficients (SCC) between gene expression profiles of primary tumor samples and each preclinical model, using different sets of the most variable genes. As shown in Figure 1C, the transcriptomic resemblance of models to primary tumors was dependent on the gene set size. At smaller gene subsets (e.g., top 100–1,000 genes), a clear hierarchy of similarity was evident, with PDX models exhibiting the highest correlation, followed by PDO and PDXO, while cell lines showed the lowest correlation. As the number of genes included in the analysis increased, the distinction between the top 3 models — PDX, PDO, and PDXO — began to shrink, showing a convergence in their global transcriptional profiles (Figure 1D). Tumor type–stratified Spearman correlation analyses for BRCA, COAD, OV, and pancreatic adenocarcinoma (PAAD) shows that the model similarity hierarchy is preserved across 4 distinct cancer types with different biology, reducing the likelihood that the findings are dominant by a single strong tissue signature (Supplemental Figure 3). Despite this convergence among the advanced models, the cell lines consistently maintained the lowest correlation across the tested model platforms.

### High stability of preclinical models across sequential passages and in vitro to in vivo transitions.

**Figure 2:**
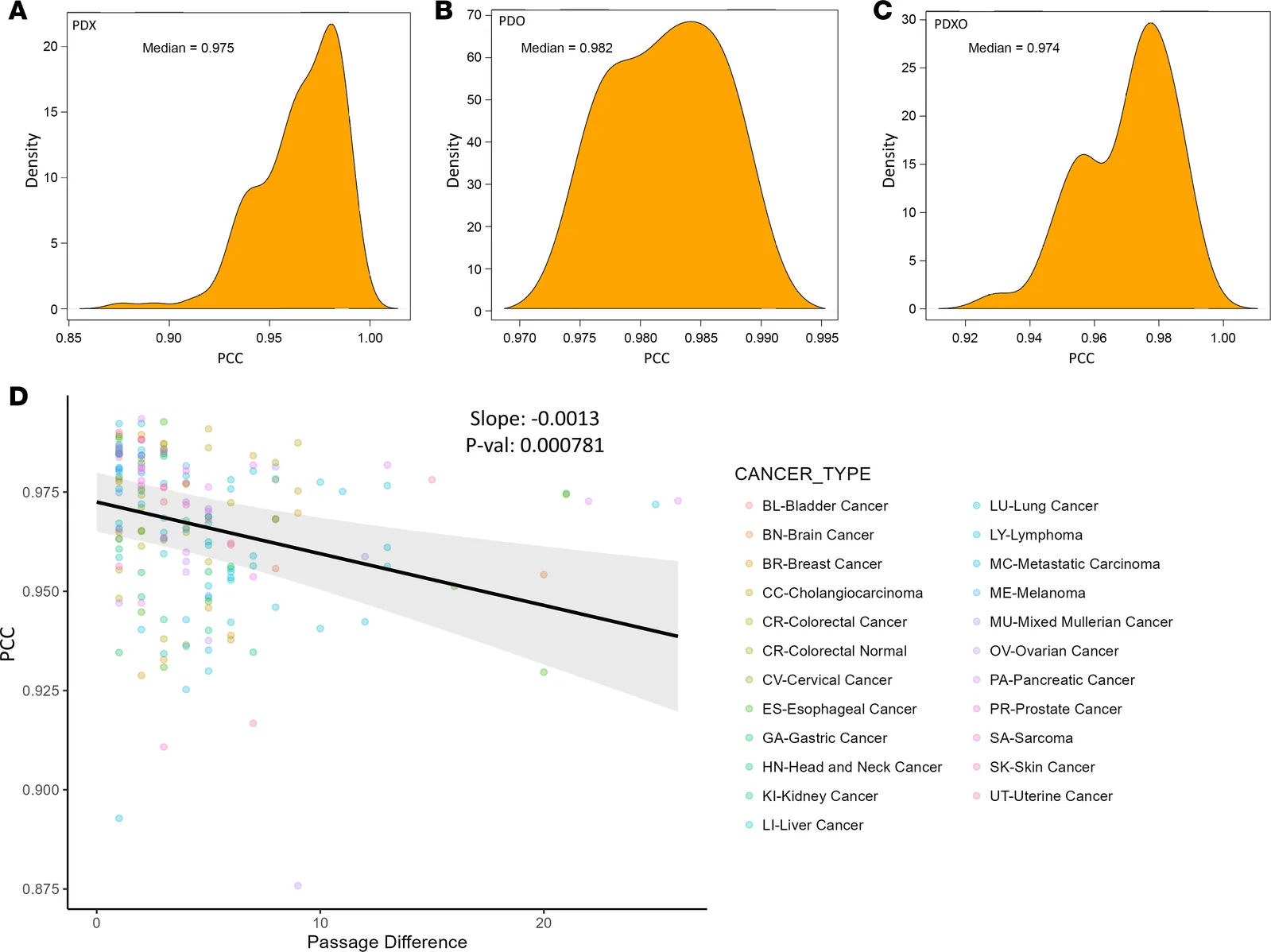
Longitudinal transcriptomic stability and passage-dependent drift. (**A**–**C**) Density plots illustrating the distribution of Pearson correlation coefficients (PCC) between different passages for PDX (**A**), PDO (**B**), and PDXO (**C**) models. The median PCC values remain high (> 0.97) across all model types. (**D**) Linear regression analysis showing the relationship between PCC and the difference in passage number between compared samples. Points are colored by cancer type. The shaded region represents the 95% CI of the fitted model.

To evaluate the longitudinal stability of the preclinical models, we assessed the transcriptomic consistency across different passages for PDX, PDO, and PDXO models. The median Pearson correlation coefficients (PCC) for these models were 0.975, 0.982, and 0.974, respectively (Figure 2, A–C). By fitting a linear model to the data, we examined the relationship between transcriptomic similarity and the difference in passage number between compared samples. Our analysis revealed a significant negative correlation (slope, –0.0013; *P* = 0.000781), demonstrating that the transcriptomic profile of a model gradually diverges as the passage gap increases (Figure 2D). This trend was observed across multiple cancer types, suggesting that a small but measurable “transcriptomic drift” occurs during long-term model maintenance, which provides a quantitative explanation for the observed variance.

**Figure 3:**
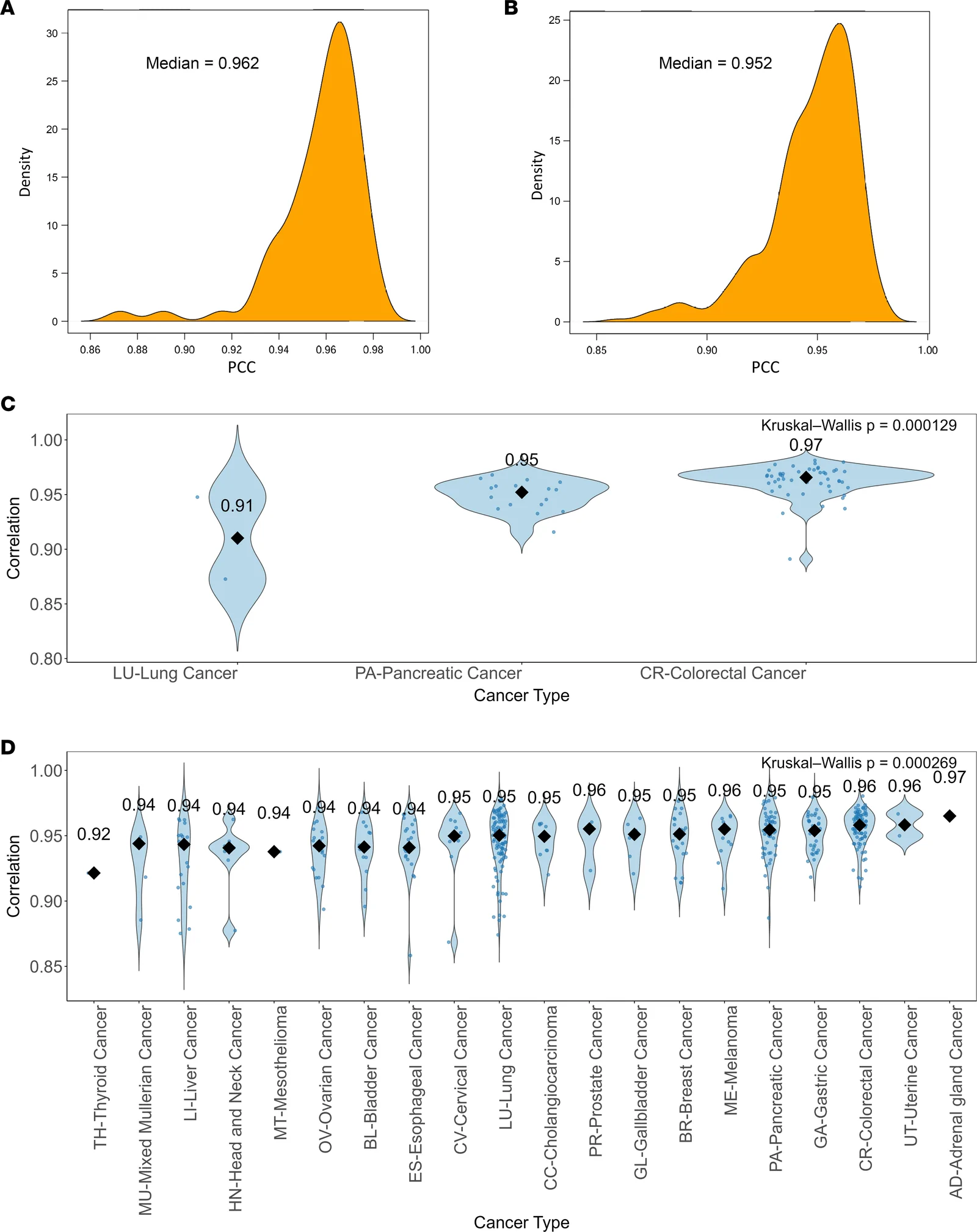
Transcriptomic conservation across patient-derived model transitions. (**A** and **B**) Density plots showing the distribution of Pearson correlation coefficients (PCC) for paired models during platform transitions: PDO versus paired PDOX (**A**) and PDX versus paired PDXO (**B**). (**C** and **D**) Violin plots representing the distribution of PCC scores stratified by cancer type for PDO vs. PDOX (**C**) and PDX vs. PDXO (**D**). Black dots indicate the median correlation for each group. Significant differences in model conservation across cancer types were observed (Kruskal-Wallis test results indicated for each).

To further evaluate the translational potential and plasticity of these models, we performed cross-platform transcriptomic comparisons between paired patient-derived systems. Specifically, we examined the correlation between PDO models and their corresponding PDOX (PDO-derived xenograft) counterparts, as well as PDX models and their paired PDXO derivatives. Our analysis revealed high transcriptomic conservation across these transitions. The median PCC between PDO and PDOX models was 0.962 (Figure 3A), while the median PCC between PDX and PDXO models was 0.952 (Figure 3B). Despite this high overall similarity, we observed significant variation in molecular similarity when stratified by tissue of origin. A Kruskal-Wallis test demonstrated that the degree of transcriptomic conservation differs significantly across cancer types for both PDO-PDOX (*P* = 0.000129; Figure 3C) and PDX-PDXO transitions (*P* = 0.000269; Figure 3D). For instance, colorectal cancer (CR) models exhibited particularly high stability during these transitions compared with other types.

### High functional similarity of preclinical models at the pathway level.

**Figure 4:**
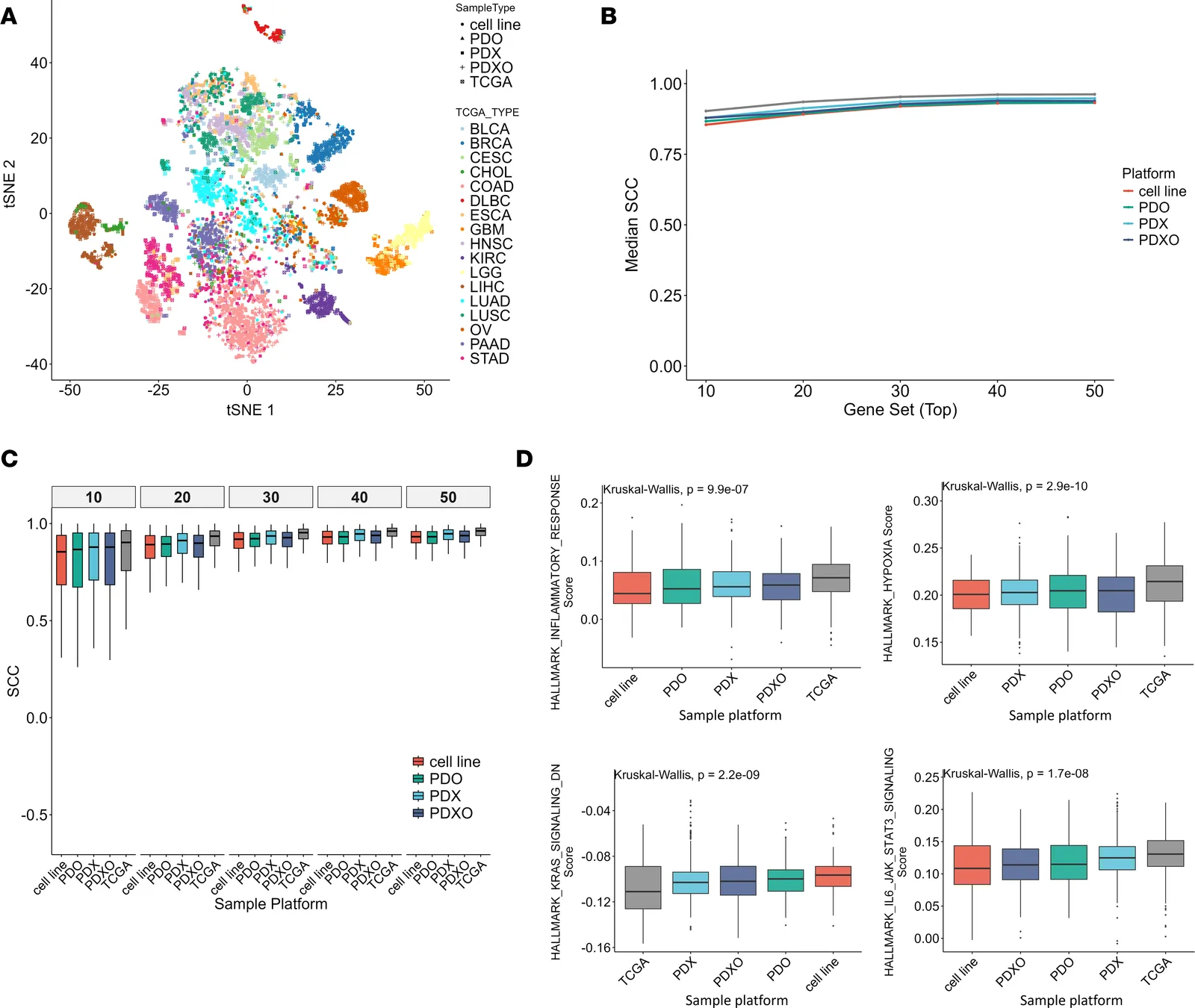
Functional transcriptomic landscape and pathway enrichment similarity. (**A**) t-SNE visualization of all samples based on ssGSEA enrichment scores for the entire MSigDB collection. Samples are colored by TCGA cancer type and shaped by model platform. (**B**) Median Spearman correlation coefficient (SCC) between preclinical models and TCGA tumors as a function of the number of top-ranked Hallmark pathways. (**C**) Box plots showing the distribution of SCC across different pathway subsets (top 10–50), stratified by model platform. (**D**) Comparison of ssGSEA enrichment scores for 4 representative Hallmark pathways: Inflammatory Response, Hypoxia, KRAS Signaling Down, and IL-6/JAK/STAT3 Signaling (Kruskal-Wallis test results indicated for each).

To determine if the transcriptomic similarity observed at the gene level translates to functional biological processes, we performed ssGSEA. We first visualized the global functional landscape using the entire MSigDB collection (32, 33) (34,808 gene sets). The t-SNE projection of these enrichment scores showed that preclinical models continue to cluster closely with their primary TCGA tumor counterparts, suggesting that the underlying biological states are well preserved across platforms (Figure 4A). We then narrowed our focus to the Hallmark gene set collection, which represents 50 well-defined biological states and processes. Similar to our previous gene-level analysis, we calculated the SCC between models and TCGA tumors across different numbers of top-ranked pathways. Across all levels of pathway complexity, from the top 10 to all 50 Hallmark sets (34), the models exhibited remarkably high and stable correlations (Figure 4B). At the functional level, the performance gap between models narrowed markedly; however, the established hierarchy remained largely intact, with PDX models consistently showing the highest functional resemblance to patient tumors (Figure 4C). We then examined representative pathways critical to tumor biology, including Inflammatory Response, Hypoxia, KRAS Signaling, and IL-6/JAK/STAT3 Signaling (Figure 4D). For these hallmarks, the distribution of enrichment scores in PDO, PDX, and PDXO models closely mirrored the distribution seen in TCGA samples. While cell lines also followed the general trend, they often exhibited wider variance or slight shifts in median scores compared with the patient-derived models.

To identify systematic pathway-level gaps between preclinical models and primary tumors, we computed the difference in median ssGSEA enrichment scores for each of the 50 Hallmark pathways. Across all 4 model types, the most consistently underrepresented programs relative to TCGA included epithelial-mesenchymal transition (EMT), angiogenesis, and WNT/β-catenin signaling (Supplemental Figure 4), which are predominantly regulated by signals from the tumor microenvironment.

### Proteomic similarity between preclinical models and primary tumors across different cancer types.

**Figure 5:**
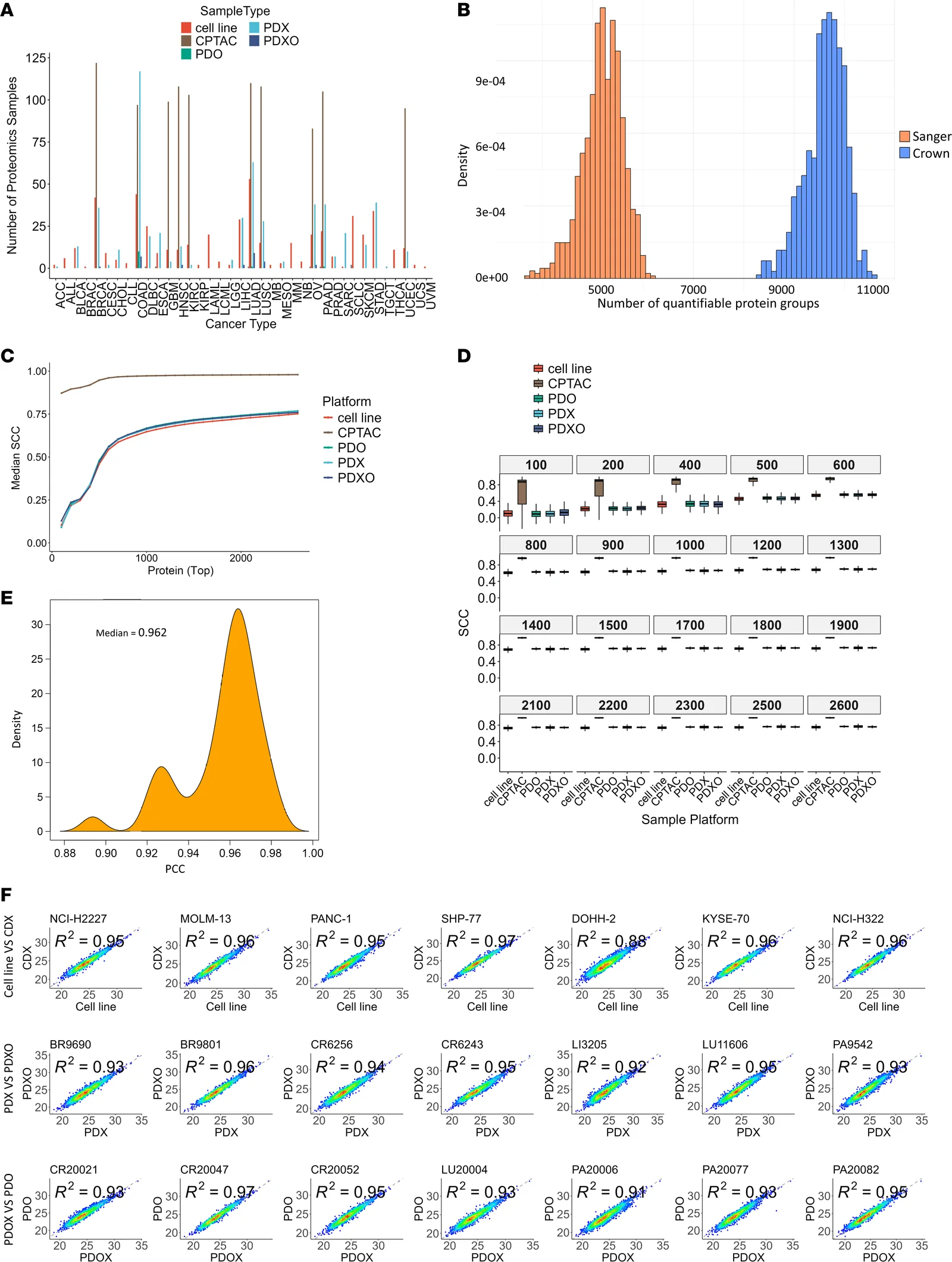
Pancancer proteomics analysis of different preclinical models. (**A**) Number of proteomics samples used, grouped by different model types and cancer types. (**B**) Density plot of number of quantifiable protein groups per sample from Sanger Institute and Crown Bioscience. (**C**) Plot of median spearman correlation coefficient between CPTAC and preclinical models against the number of top varied proteins. (**D**) Box plot of spearman correlation coefficient between proteomics samples of CPTAC and preclinical models for each model type, each panel represents the corresponding top *n* varied proteins of CPTAC samples. (**E**) Density plots showing the distribution of Pearson correlation coefficients (PCC) for paired in vitro and in vivo models during platform transitions. (**F**) Spearman correlations between proteomics of cell line and CDX (upper panel), between PDX and PDXO (middle panel), and between PDOX and PDO (lower panel).

We compared proteomic profiles from 1,030 primary tumors from CPTAC with 595 PDX samples, 29 PDXO samples, 12 PDO samples, 7 cell line-derived xenograft (CDX) samples, and 643 cell lines from Crown Bioscience collection, covering 37 tumor types (Figure 5A). The protein expression data obtained from Crown Bioscience were normalized using the retention time–dependent normalization method implemented in DIA-NN v1.9.2 (35), and the batch effects associated with different model types were corrected using ComBat (31).

We first validated the depth of the Crown Bioscience dataset by comparing it against the Sanger Institute’s cell line proteomic data. The Crown dataset exhibited significantly deeper coverage, with a median of 9,666 quantifiable protein groups per sample compared with 5,220 in the Sanger dataset (Wilcoxon rank-sum test, *P* < 0.0001; Figure 5B), confirming the high quality and analytical depth of our primary data source.

Following the framework of our transcriptomic analysis, we evaluated the proteomic similarity between preclinical models and primary tumors using varying sets of the most variable proteins. At lower protein set sizes, all models exhibited relatively low correlations to CPTAC tumors. However, as the analysis expanded to include more proteins, a distinct hierarchy emerged: PDX models displayed the highest similarity to primary tumors, followed closely by PDXO and PDO models, which performed nearly identically, while cell lines consistently showed the lowest correlations (Figure 5, C and D). Although the absolute correlation coefficients were lower than those at the transcriptomic level, the relative order of resemblance (PDX > PDXO = PDO > cell line) remained consistent across both omics layers.

Finally, we assessed the proteomic stability during in vitro to in vivo transitions by comparing matched pairs, including cell line–CDX, PDX-PDXO, and PDOX-PDO pairs. Strong proteomic conservation was observed across these transitions, with a median PCC of 0.962 (Figure 5E). Individual scatter plots for these transitions further illustrate the tight correlation of protein expression levels, regardless of the model platform (Figure 5F).

### WES outperforms RNA-seq in mutation detection.

**Figure 6:**
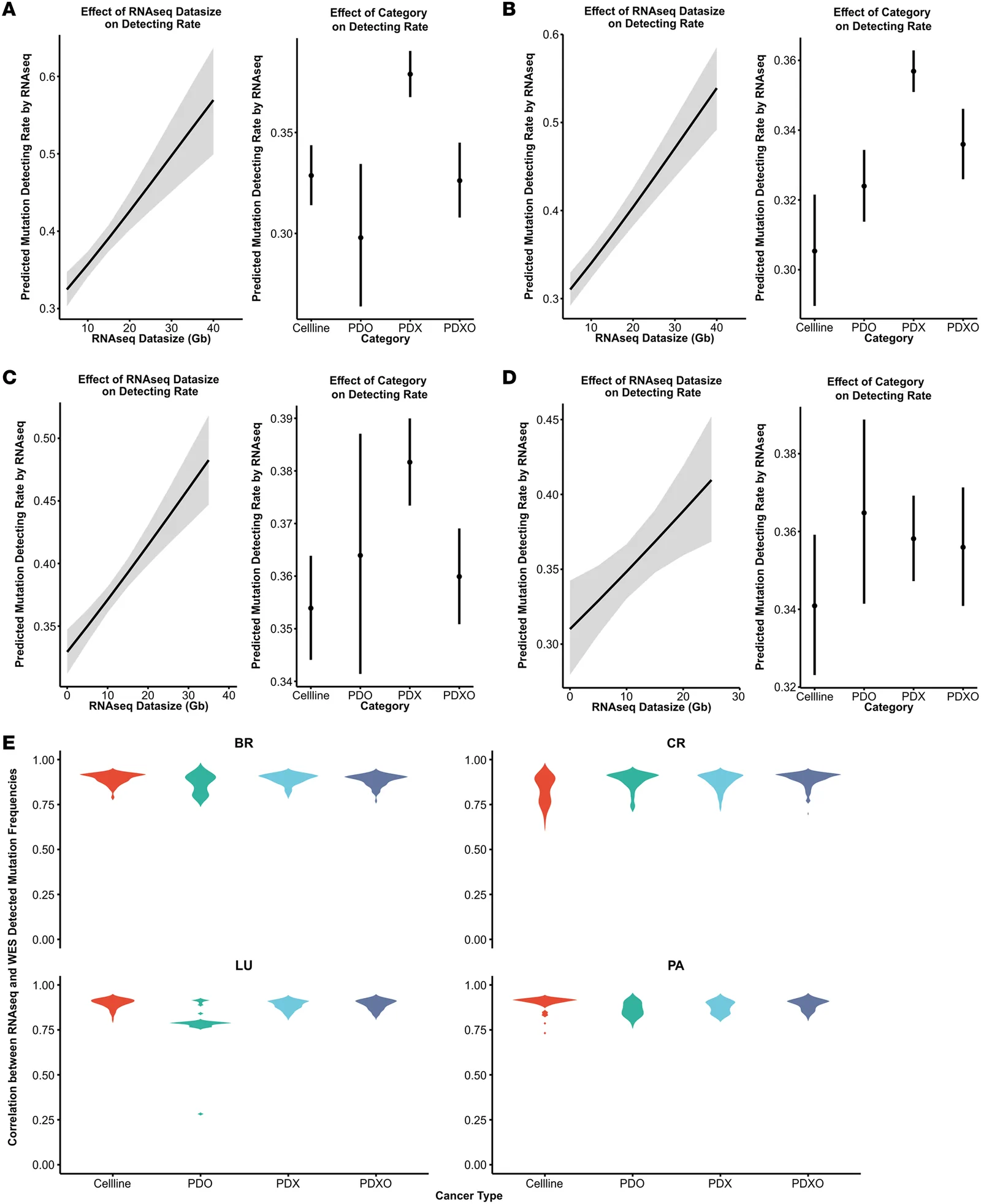
Mutation detection by WES and RNA-seq from in vitro and in vivo tumor models. (**A**–**D**) Predicted detection rate by Beta regression model with RNA-seq data size and model category in 4 cancer types: BR, CR, LU, and PA. (**E**) Spearman correlation coefficient on mutation frequencies estimated by RNA-seq and WES in 4 cancer types.

Whole-exome sequencing (WES) and whole-transcriptome sequencing (WTS, RNA-seq) are powerful tools for detecting single-nucleotide variants (SNVs) at the DNA and RNA level, respectively. To systematically compare their mutational detection capabilities, we performed WES and RNA-seq on a large cohort of 3,179 preclinical human tumor models, comprising 692 cell lines, 176 PDOs, 1,748 PDXs, and 563 PDXOs. The median sequencing data size, which is a measure of sequencing depth, is 15.18 Gb (approximately 125 average sequencing depth) for WES, 12.74 Gb for RNA-seq. For each model, we identified high-quality SNVs from both datasets. We then calculated the fraction of RNA-seq–derived mutations detectable by WES and vice versa. The vast majority (94.7%–95.1%) of RNA-seq–based SNVs were also identified by WES. In contrast, the average RNA-seq detection rate for WES-based mutations was lower (33.2%–37.2%), indicating that over 60% of genomic variants are undetectable after transcription (Supplemental Figure 5). To control for confounding variables, we employed a beta regression model to assess the effect of RNA-seq data size and model category on the RNA-seq detection rate within 4 major cancer types: breast (BR), colorectal (CR), lung (LU), and pancreatic (PA) (Figure 6, A–D). Despite the difference in detection rates, the mutation frequencies inferred by WES and RNA-seq were highly consistent across these cancers, with high mean SCC (BR: 0.890, CR: 0.874, LU: 0.884, PA: 0.882) (Figure 6E).

### Panmodel and pancancer comparison of intratumor heterogeneity.

**Figure 7:**
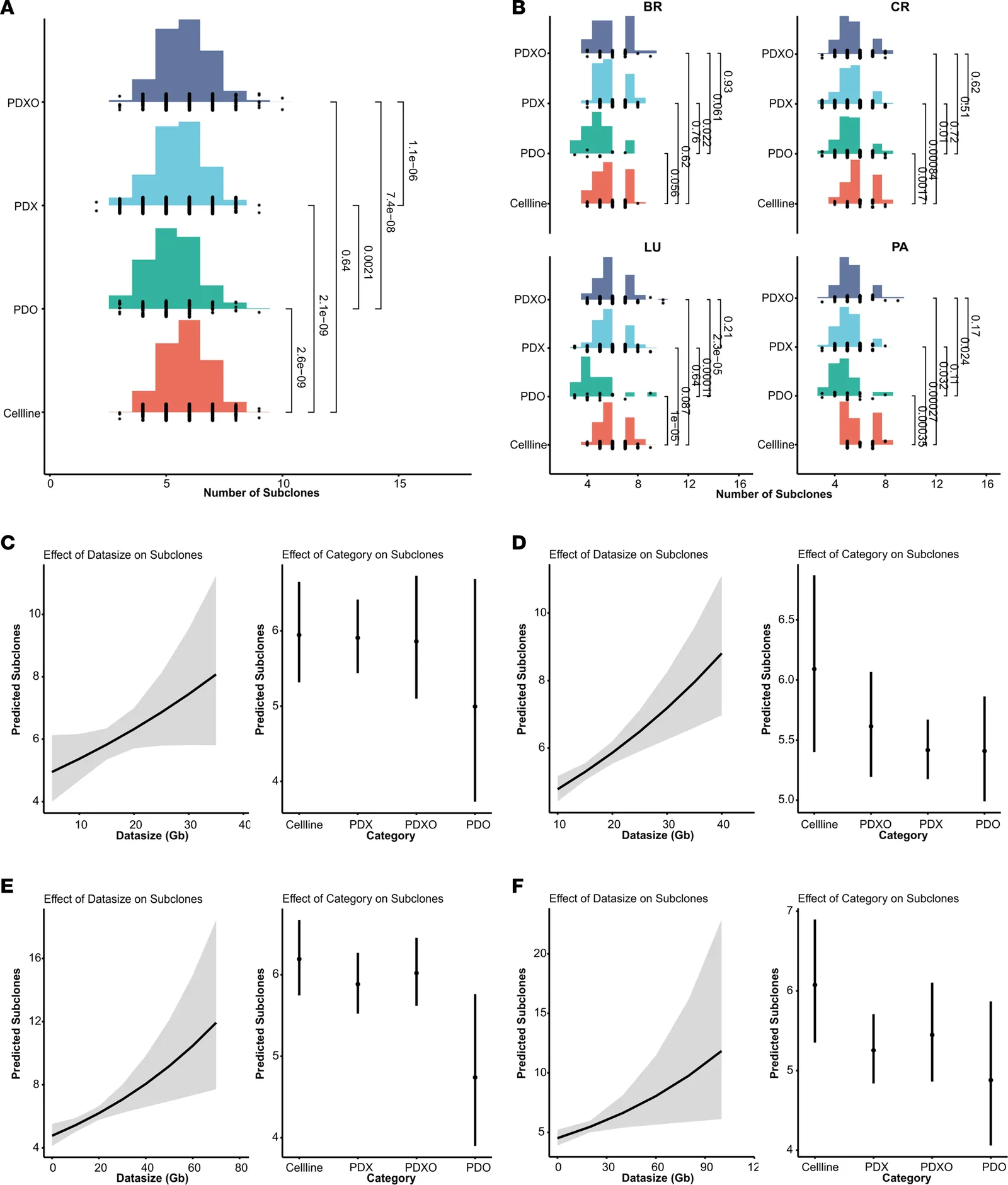
Tumor clonality prediction from WES data across tumor model systems. (**A**) Histograms of subclone counts inferred from cell line, PDO, PDX, and PDXO tumor samples. (**B**) Histograms of subclone counts inferred from 4 model types on 4 cancer types: BR, CR, LU, and PA. (**C**–**F**) Predicted number of subclones by Poisson regression model with WES data size (left panel) and model category (right panel) in 4 cancer types: BR (**C**), CR (**D**), LU (**E**), and PA (**F**).

Intratumor heterogeneity (ITH) is a crucial factor in tumor development and therapeutic response. Computational methods infer tumor subclones from bulk sequencing data such as whole genome sequencing and WES by analyzing variant allele frequencies (VAF) to deconstruct its mixed cell population. We used WES data for 4,690 tumor models (740 cell lines, 207 PDOs, 3,075 PDXs, and 668 PDXOs) and estimated tumor clonality by using mutation frequency information for each tumor sample. Analysis of subclone counts revealed significant differences across model types (Wilcox rank-sum test *P* < 0.05), with a consistent hierarchy of clonal complexity: cell lines > PDXOs > PDXs > PDOs (Figure 7A). This trend was largely conserved across the BR, CR, LU, and PA cancer types (Figure 7B). Since sequencing depth is known to influence VAF variance and subclone estimation — i.e., deeper sequencing results in smaller predicted tumor heterogeneity (36) — we verified the findings by controlling the WES data sizes. Specifically, we built a Poisson regression model adjusting for WES data size and model category. This analysis confirmed that subclone count estimates were positively correlated with sequencing depth; furthermore, after adjusting for data size, cell lines — particularly those with high passage numbers — exhibited the most complex ITH (Figure 7, C–F).

## Discussion

In this study, we conducted a systematic, multi-omic comparison of transcriptomic, proteomic, and genomic profiles across 4 major preclinical cancer models — cell lines, PDXs, PDXOs, and PDOs — benchmarking them against primary tumor samples from TCGA and CPTAC. Our objective was to establish a quantitative hierarchy of molecular similarity and evaluate how platform transitions and long-term passaging affect model stability. Through the large-scale integration of these datasets, we demonstrated that, while all models generally recapitulate their tissue of origin, a distinct hierarchy of resemblance exists: PDX > PDO = PDXO > cell line.

A key finding of our analysis is the high degree of transcriptomic and proteomic conservation during in vitro to in vivo transitions. The robust Pearson correlations observed during model interconversion indicate that the core molecular programs remain largely intact across different growth environments. Our pathway-level analysis further reinforces these findings. The results indicate that patient-derived preclinical models not only maintain global transcriptional similarity but also accurately recapitulate the functional pathway activities characteristic of human cancers. This functional robustness, observed across the Hallmark gene set collection, suggests that, even when individual gene expression fluctuates, the underlying biological processes remain well-preserved and stable.

At the transcriptomic level, preclinical models preserve the major transcriptomic signatures of their corresponding cancer types. Well-defined clusters for common tumors such as BR, LU, CR, and ovarian cancers suggest strong representativeness and low intramodel variability. However, several tumor types — including cholangiocarcinoma (CHOL), diffuse large B cell lymphoma (DLBC), cervical squamous cell carcinoma (CESC), and esophageal carcinoma (ESCA) — displayed more diffuse or sparse clustering patterns. This dispersion may reflect biological heterogeneity within these cancers, differences in tumor purity, or the smaller sample sizes available for certain cancer types (37–40). Moreover, tumors with complex stromal or immune components often show greater transcriptomic variability (41), which can lead to less distinct clustering in both patient tumors and derived models.

Interestingly, the same order of model similarity was observed at both transcriptomic and proteomic levels, suggesting that these trends reflect intrinsic biological association rather than platform-specific artifacts. Nonetheless, the overall correlation between preclinical models and primary tumors was consistently lower at the proteomic level. The mechanisms driving this reduction remains to be elucidated and is likely caused by posttranscriptional and posttranslational regulatory mechanisms which are known to decouple protein abundance from transcript levels (42, 43). While the proteomic hierarchy aligned perfectly with the transcriptomic findings, the sample sizes for advanced in vitro models are relatively small compared with the broader cohort. Future large-scale proteomic profiling is warranted to further validate these specific platform transitions.

From a genomic perspective, in the context of small variant detection, WES at the DNA level demonstrates a superior detection rate compared with RNA-seq across all tumor model platforms. Small fractions of RNA-seq unique SNVs were primarily due to their location out of the WES-capture boundary regions, as well as low coverage of the relevant regions or RNA editing (36). We observed that the RNA-seq–based detection rate for WES-identified mutations was moderately higher in PDXs than in PDXOs, PDOs, and cell lines. This discrepancy may be attributed to the presence of murine stromal content in xenografts, which can lead to an overestimation of truly detectable human tumor mutations. In contrast, RNA-seq analysis of cell lines and PDOs, which comprise pure human tumor cells, detected only approximately 33.2% of the mutations identified by WES. For tumor clonality analysis, and irrespective of systematic biases introduced by WES sequencing depth, we hypothesize that ITH is strongly influenced by the duration of passaging following the initial culture of primary tumor tissues. Cell lines, which are long-term in vitro models passaged extensively over numerous generations, exhibited the highest degree of heterogeneity. This is consistent with sufficient time for evolutionary divergence within the tumor cell population. PDOs, established directly from patient tumors and typically passaged a median of 7 times, showed less heterogeneity. PDXs, as in vivo models, underwent 0–25 passages (median, 3). PDXOs are derived from PDX tumors after 6–10 passages during their establishment. Consequently, PDXOs displayed greater subclonal diversity than their parent PDXs, reflecting continued evolution under in vitro culture conditions.

Altogether, this comprehensive multi-omic benchmark provides a vital, data-driven framework for navigating the complex landscape of preclinical cancer models. By defining the hierarchy of molecular similarity and illuminating the effect of prolonged passaging on ITH, these insights empower researchers to select platforms that optimally balance biological representativeness with experimental practicality. Ultimately, deploying the right model for the right experimental goal is essential for bridging the translational gap and improving the clinical success rate of precision oncology therapeutics.

Our findings are broadly concordant with the existing literature. The observation that cell lines show the lowest molecular similarity to primary tumors is consistent with high-grade serous ovarian cancer–specific (HGSOC-specific) comparisons, which document systematic transcriptomic and genomic shifts in cell lines relative to patient tumors (8). The high molecular similarity of PDX models is consistent with the large-scale PDX genomic characterization by Gao et al. (13) and the systematic review by Byrne et al. (44). The principal contribution of our study relative to these prior works is the systematic, multiplatform, multi-omic quantification of this hierarchy, within a unified analytical framework that has not previously been applied across all 4 major preclinical cancer model platforms simultaneously.

This study has several additional limitations. All preclinical model data were generated at Crown Bioscience, introducing the possibility of institution-specific systematic biases; independent replication using external model collections will be important to confirm the generalizability of these findings. The removal of the murine stromal compartment from the RNA-seq and proteomics data of PDX models means that the similarity metrics reported in this model platform mainly focus on the human tumor cell compartment, and microenvironmental similarity is left to be assessed. The sample sizes for advanced in vitro models in the proteomic analysis (PDO, *n* = 12; PDXO, *n* = 29) are limited, and the proteomic conclusions for these categories should be interpreted with caution. The usage of global correlation coefficients to evaluate the model similarities to clinical samples is a summary statistic that does not capture locus-specific or functionally critical divergences. Future analyses incorporating gene-level outlier detection or pathway-specific concordance metrics would complement this global framework.

## Methods

### Sex as a biological variable.

In this study, patient-derived models (including PDXs, PDXOs, PDOs, and cell lines) as well as public dataset cohorts originated from both male and female patients. Sex was not considered as a biological variable.

### Model establishment and public datasets.

Methods and parameters regarding establishment of preclinical models have been described previously (45–47). Reference patient data were obtained from TCGA for transcriptomics and CPTAC for proteomics. Standardized cell line data were retrieved from the Cancer Cell Line Encyclopedia (CCLE) (48) and the Genomics of Drug Sensitivity in Cancer (GDSC) websites (49). To ensure comparability across diverse sources, batch effects associated with different study origins and model platforms were corrected using the ComBat algorithm with default parameters (31). All PDXs, PDXOs, PDOs, CDXs, and associated host animals utilized for sequencing in this study were provided by Crown Bioscience. Cell line models were sourced from the Crown Bioscience collection as well as the CCLE.

### Bulk RNA-seq and data processing.

Total RNA was extracted from preclinical models using the RNeasy Mini Kit (Qiagen). RNA quantity and quality were assessed via NanoDrop 2000 (Thermo Fisher Scientific) and Agilent 4200 TapeStation. Libraries were constructed using the TruSeq RNA Sample Prep Set (Illumina) following the standard protocol: poly-A selection via oligo-dT beads, fragmentation, cDNA synthesis, end repair, A-tailing, and adapter ligation. Final libraries were quantified using a Qubit 3.0 fluorometer (Thermo Fisher Scientific), analyzed for size distribution on a TapeStation, and sequenced on the Illumina NovaSeq 6000 platform.

Raw data quality was assessed using FastQC v0.11.3 (50), with adapters and low-quality sequences trimmed by Trimmomatic v0.36 (51). To distinguish species in mixed-species samples, cleaned reads were mapped to human (hg19) and mouse (mm10) reference genomes using STAR v2.5.1b (52). Reads mapping to both genomes were assigned to the species with fewer alignment mismatches. Gene expression was quantified via pseudoalignment using Kallisto v0.42.5 (53) against the UCSC GENCODE V24 Basic (human) and mm10 (mouse) annotations. All reads identified as originating from the mouse host were explicitly removed prior to proceeding with the downstream expression analysis.

### WES and data processing.

Genomic DNA was extracted using the KingFisher Flex system with the MagMAX DNA Multi-Sample Ultra 2.0 Kit. High-quality DNA (concentration > 100 ng/μL; A_260_/A_280_ ≥ 1.8) was fragmented using an ME220 Focused-ultrasonicator (Covaris). Libraries were prepared using the SureSelect XT HS2 DNA Reagent Kit (Agilent) according to the manufacturer’s instructions, involving end repair, dA-tailing, adapter ligation, and PCR amplification with AMPure XP bead purification. Final libraries were sequenced (150 bp paired-end) on the NovaSeq 6000 platform.

Bioinformatic processing followed a similar pipeline to RNA-seq for quality control (FastQC, Trimmomatic). Cleaned reads were aligned to hg19 and mm10 genomes using BWA v0.7.12 (54); reads preferentially mapping to the mouse genome were discarded.

### Proteomic sequencing and data processing.

To prepare preclinical models for proteomic analysis, host murine stromal cells were depleted from xenograft models (PDX and CDX) using the Human Tumor Dissociation Kit (Miltenyi Biotec) and a gentle MACS Dissociator (Miltenyi Biotec, A19337). The resulting single-cell suspensions were incubated with a Mouse Cell Depletion Cocktail at 2°C–8°C for magnetic labeling. Labeled host cells were retained on an LS column within a MACS Separator, while enriched human tumor cells were collected from the flow-through, centrifuged a 300*g* for 5 min, and stored as pellets of 5 × 10^6^ cells at –80°C. This depletion step was bypassed for cell lines and organoids (PDOs and PDXOs), which proceeded directly to subsequent steps. For sequencing, approximately 5 million cells were resuspended in 500 μL lysis buffer (8M urea, 50 mM ABC, 1 mM DTT) with protease and phosphatase inhibitors, before being sonicated on ice. Following BCA protein quantification, 500 μg of lysate was reduced with 5 mM DTT at 37°C for 2 hours and alkylated with 10 mM iodoacetamide in the dark for 45 minutes. Samples were diluted to 1M urea and digested with trypsin (1:50 w/w) for 16 hours at 37°C. Digestion was halted with 20% formic acid (pH < 2) before peptides were desalted using MonoSpin or Sep-Pak C18 columns and vacuum dried. Peptide separation and analysis were performed using a Vanquish Neo ultra-high-performance liquid chromatography system interfaced with an Orbitrap Astral mass spectrometer (Thermo Fisher Scientific). Approximately 300 ng of peptides were loaded onto an Easy-Spray C18 column (150 μm × 15 cm) and fractionated via a 24-minute gradient elution under data-independent acquisition (DIA) mode. Peptides were ionized through electrospray ionization (2.0 kV) with an inlet capillary temperature of 280°C and an ion-funnel RF of 40%. MS1 survey scans were acquired in the Orbitrap at a resolving power of 240,000 (*m/z* 380–980) with a 0.6-second cycle time. For MS/MS, precursor isolation windows were set to 2 Th (nonoverlapping) across *m/z* 380–980. HCD fragmentation was performed at a normalized collision energy of 25%, and spectra were acquired in the Astral analyzer (*m/z* 150–2000) with a maximum injection time of 3 ms and an AGC target of 500%.

Raw Orbitrap files were processed using DIA-NN v1.9.2 (35) in library-free mode against the human reviewed proteome. A 2-pass workflow was employed, first generating an empirical spectral library from DIA runs to reanalyze the data. Database searching utilized Trypsin/P as the protease, allowing for 1 missed cleavage. Fixed modifications included carbamidomethyl (C), while variable modifications included methionine (Met) oxidation, N-terminal Met excision, and protein N-terminal acetylation. Match between runs (MBR) was enabled, and outputs were filtered at a 1% FDR. Quantified protein data were log transformed to achieve an approximate normal distribution for downstream statistical analysis. Missing values were addressed either through listwise deletion for quantitative comparisons or via data imputation using the impSeqRob algorithm (55) (rrcovNA R package) for dimension reduction. Batch effect correction was performed using the ComBat algorithm with default parameters.

### Algorithm for calculating SCC between preclinical models and primary tumors.

To quantify the molecular similarity of preclinical models relative to primary tumors, we employed a robust subsampling and correlation framework. First, the most variable features — genes, proteins, or gene sets — were identified based on their SD within the reference patient datasets, such as TCGA or CPTAC. To account for sample size imbalances across different platforms, we implemented a bootstrapping approach consisting of 100 iterations. In each iteration, we randomly sampled *n* = 20 samples per cancer type for each preclinical model platform, while a corresponding reference set of *n* = 20 patient samples per cancer type was independently sampled from the TCGA/CPTAC cohorts. For any cancer type or platform category containing fewer than 20 samples, sampling was performed with replacement to ensure robust statistical estimation. SCC were then calculated between the paired model and reference sets across a sliding scale of the top *N* most variable features — e.g., ranging from *N* = 100 to the full feature set size. Finally, the similarity scores were represented as the median correlation across all 100 iterations, a process that allowed us to observe how model similarity stabilizes or converges as the number of analyzed biological features increases.

### Mutation detection from RNA-seq and WES data.

Variants were identified using GATK v4.2.4.0 (56) HaplotypeCaller and annotated with VEP v90 (57). Variants were filtered to exclude those with an allele frequency (AF) > 0.001 in public databases or > 0.2 in Crown Bioscience’s tumor models to remove germline variants. Driver mutations were predicted via the Genome Interpreter database (58), and copy number variations (CNV) were detected using GATK.

### Integration of mutation calls.

High-confidence mutations identified from both RNA-seq and WES were compared with calculate detection rates and concordance. A beta regression model was employed to evaluate the effect of RNA-seq sequencing depth (data size) and tumor model type on the RNA-seq detection rate of WES-based mutations across 4 cancer indications: BR, CR, LU, and PA.

### Tumor clonality inference from WES data.

High-quality somatic SNV altered and reference read count data were used as input for tumor clonality prediction by MAGOS algorithm (59). A Poisson regression model was built on inferred subclone count to evaluate the effect of WES data size and tumor model category (PDO, PDX, and PDXO versus cell line) for different cohorts of cancer indications (BR, CR, LU, and PA).

### Statistics.

To quantify molecular similarity, SCC and PCC were calculated. For continuous variables compared across multiple independent groups, such as transcriptomic conservation and ssGSEA enrichment scores, a Wilcoxon rank-sum test was utilized. Differences in subclone counts between model types were evaluated using the Wilcoxon rank-sum test. Linear regression model was used to examine the relationship between transcriptomic similarity and differences in passage numbers. To adjust for covariates, the beta regression model was employed to evaluate mutation detection rates, and the Poisson regression model was utilized to analyze inferred subclone counts while controlling for WES data size. A *P* value of less than 0.05 was considered statistically significant. All statistical tests are 2 sided.

### Study approval.

Procedures for all animal studies were performed in adherence to IACUC-approved protocols at the Crown Bioscience specific pathogen–free (SPF) animal facility.

### Data availability.

Processed transcriptomic and proteomic data for primary human tumors were downloaded from public repositories, including TCGA and the CPTAC, through the Genomic Data Commons (GDC) Data Portal (https://portal.gdc.cancer.gov/) and LinkedOmicsKB (https://kb.linkedomics.org/). Processed transcriptomic, genomic, and proteomic data for preclinical models were publicly available to registered users through the Crown Bioscience Database portal with free registration (https://db.crownbio.com/). Processed transcriptomic and genomic data for publicly available cancer cell lines were retrieved from the CCLE through the DepMap portal (https://depmap.org/portal/) and the Sequence Read Archive (SRA; https://www.ncbi.nlm.nih.gov/bioproject/PRJNA523380).

All Supporting data values associated with the main manuscript and Supplemental figures are provided in the Supporting Data Values file.

## Author contributions

ZX performed data analysis and drafted the manuscript, authoring the bulk of the Results — covering expression, model stability, pathway-level function, and proteomic similarity. JX contributed to data analysis and manuscript composition, writing the remainder of the Results, on mutation detection and intratumor heterogeneity. BM, HL, and WQ assisted with data analysis. JW and XC contributed to data generation. SG designed the research, oversaw the project, and revised the manuscript.

## Conflict of interest

This research was funded by Crown Bioscience Inc., and authors affiliated with Crown Bioscience were employees when the study was performed.

## Funding support

- This research was funded by Crown Bioscience Inc.

## Supplementary Material

Supplemental data

Supporting data values

## Figures and Tables

**Table 1:**
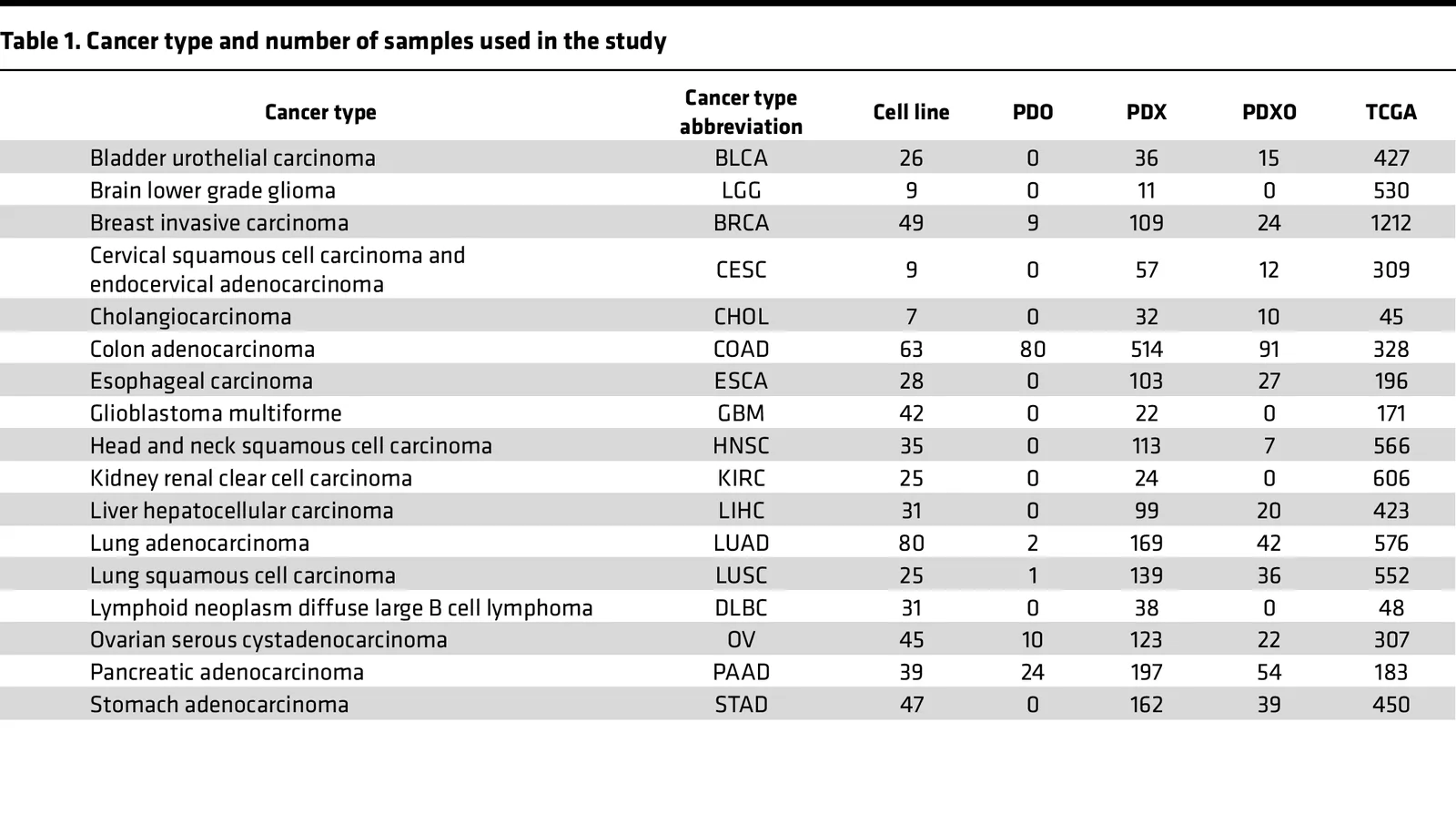
Cancer type and number of samples used in the study

## Version 1 06/30/2026

In-Press Preview

## Version 2 08/24/2026

Electronic publication
