# Supplementary material for: The molecular similarity landscape of preclinical cancer models to patient tumors
Source: JCI Insight. 2026 Jun 30;11(16):e206449. doi: 10.1172/jci.insight.206449 (PMC13502168; doi:10.1172/jci.insight.206449)
Supplement: Supplementary file 1 [file jciinsight-11-206449-s319.pdf]

The Molecular Similarity Landscape of Preclinical Cancer Models to Patient Tumors

Authors: Zixuan Xie[1], Jia Xue[1], Binchen Mao[1], Hengyuan Liu[1], Wubin Qian[1], Jingjing Wang[1], Xiaobo Chen[1], Sheng Guo[1,2]*

Affiliation:

[1]Crown Bioscience Inc., Suzhou, Jiangsu, China, 215000.

[2]Biomedical Basic Research Center (BBRC) of Jiangsu, Suzhou, Jiangsu, China, 215123.

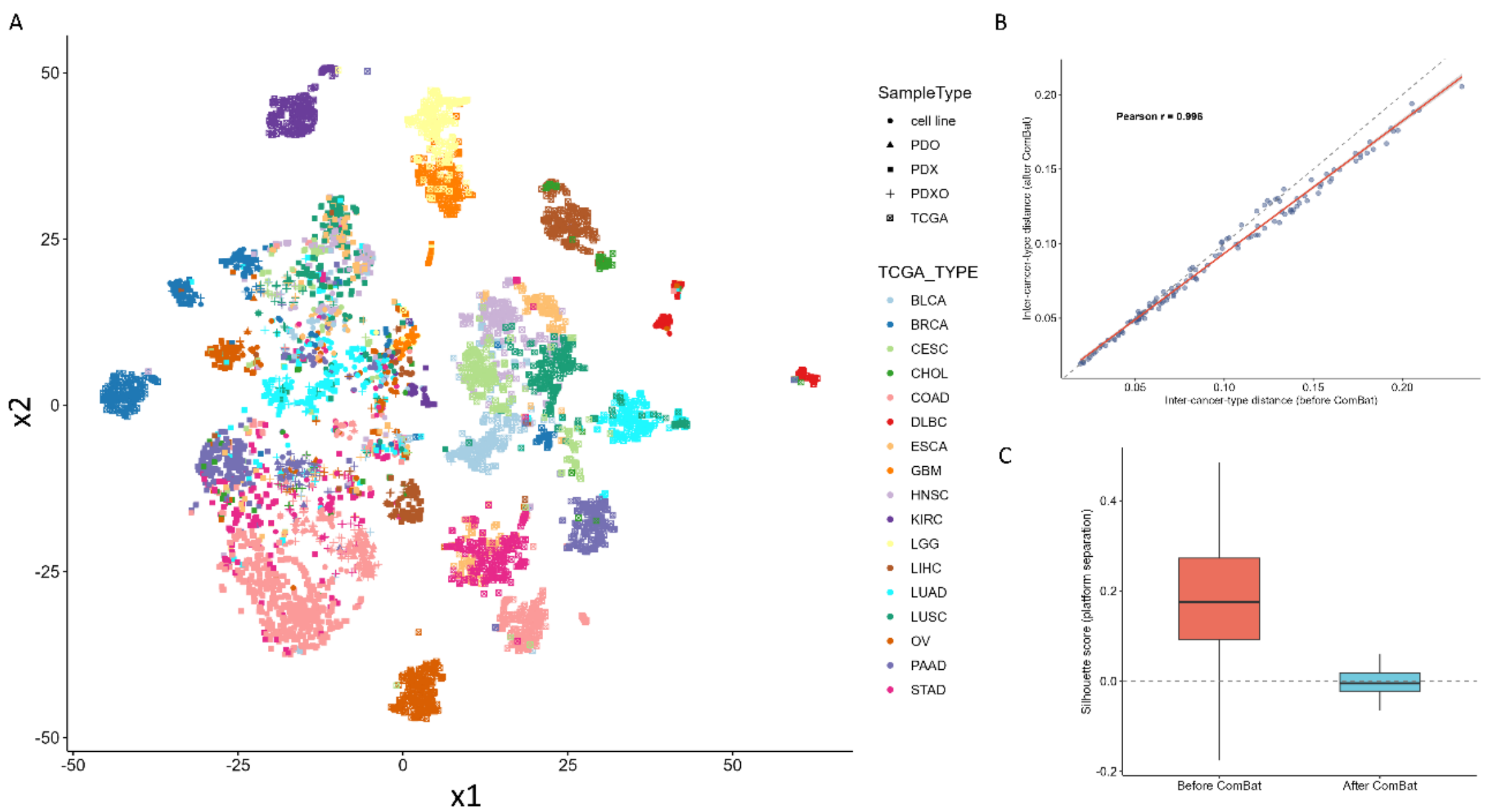

Supplementary Figure 1. Validation of ComBat batch correction. (**A**) t-SNE projection of all RNA-Seq samples without batch correction, colored by TCGA cancer type and shaped by model platform. (**B**) Scatter plot of pairwise inter-cancer-type correlation distances computed from TCGA-only centroids before (x-axis) and after (y-axis) ComBat correction, across all 136 cancer type pairs (17 cancer types). (**C**) Silhouette scores quantifying platform-based sample clustering before and after ComBat correction. A positive silhouette score indicates that a sample is more similar to other samples from the same platform than to samples from different platforms. Before correction, the median silhouette score was 0.18, reflecting substantial platform-driven clustering. After correction, the median score decreased to approximately 0, indicating that platform separation was effectively eliminated.

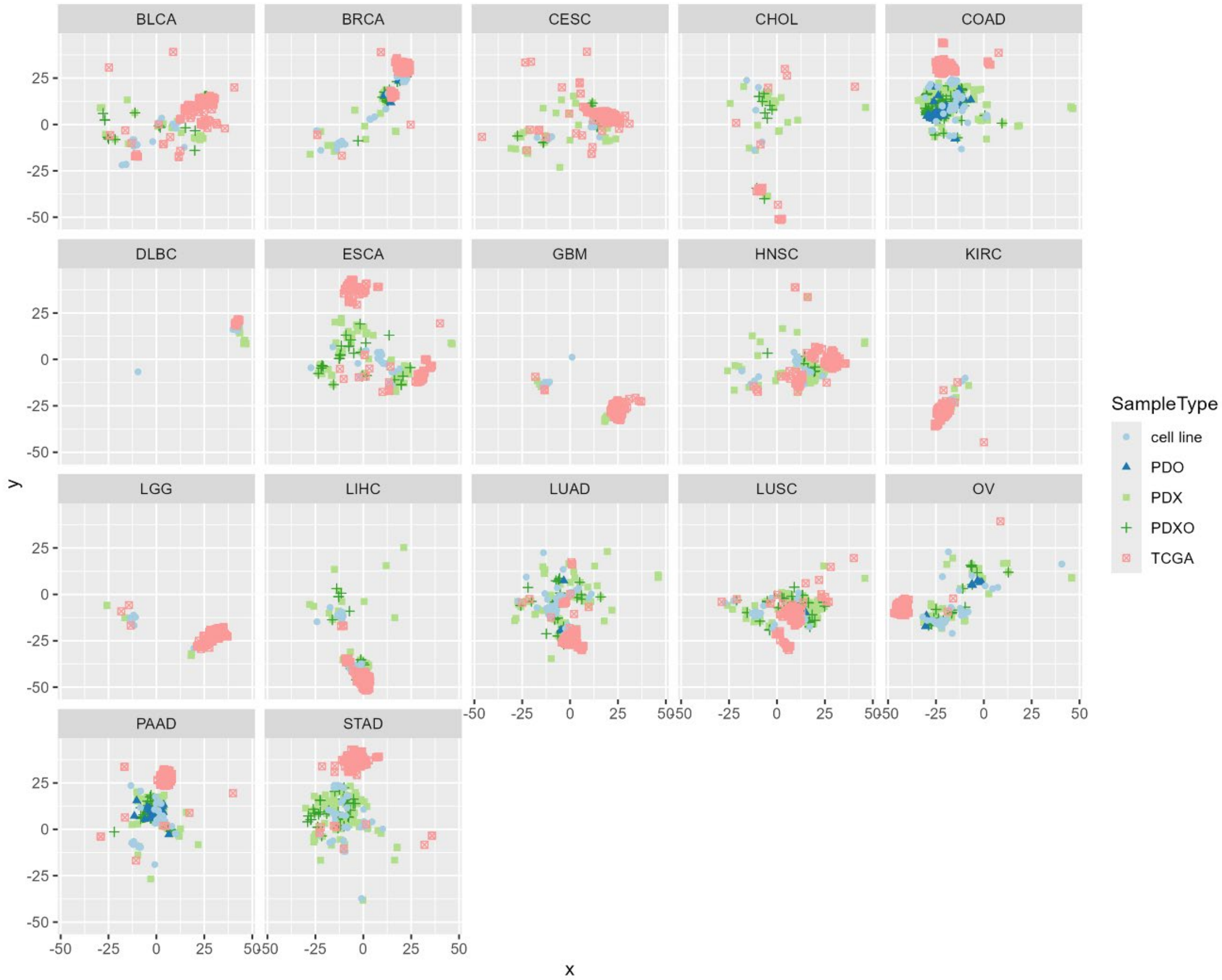

Supplementary Figure 2. Tumor type-stratified t-SNE analysis of RNA-Seq profiles across preclinical models and primary tumors. t-SNE projections of batch-corrected RNA-Seq profiles are shown separately for each of the 17 tumor types analyzed in this study. For most tumor types with sufficient model representation, preclinical models cluster in proximity to their corresponding TCGA samples, supporting the population-level molecular similarity reported in the main text. Tumor types with sparse model representation (e.g., CHOL, DLBC, GBM, LGG) or high biological heterogeneity (e.g., CESC, ESCA) show more dispersed patterns.

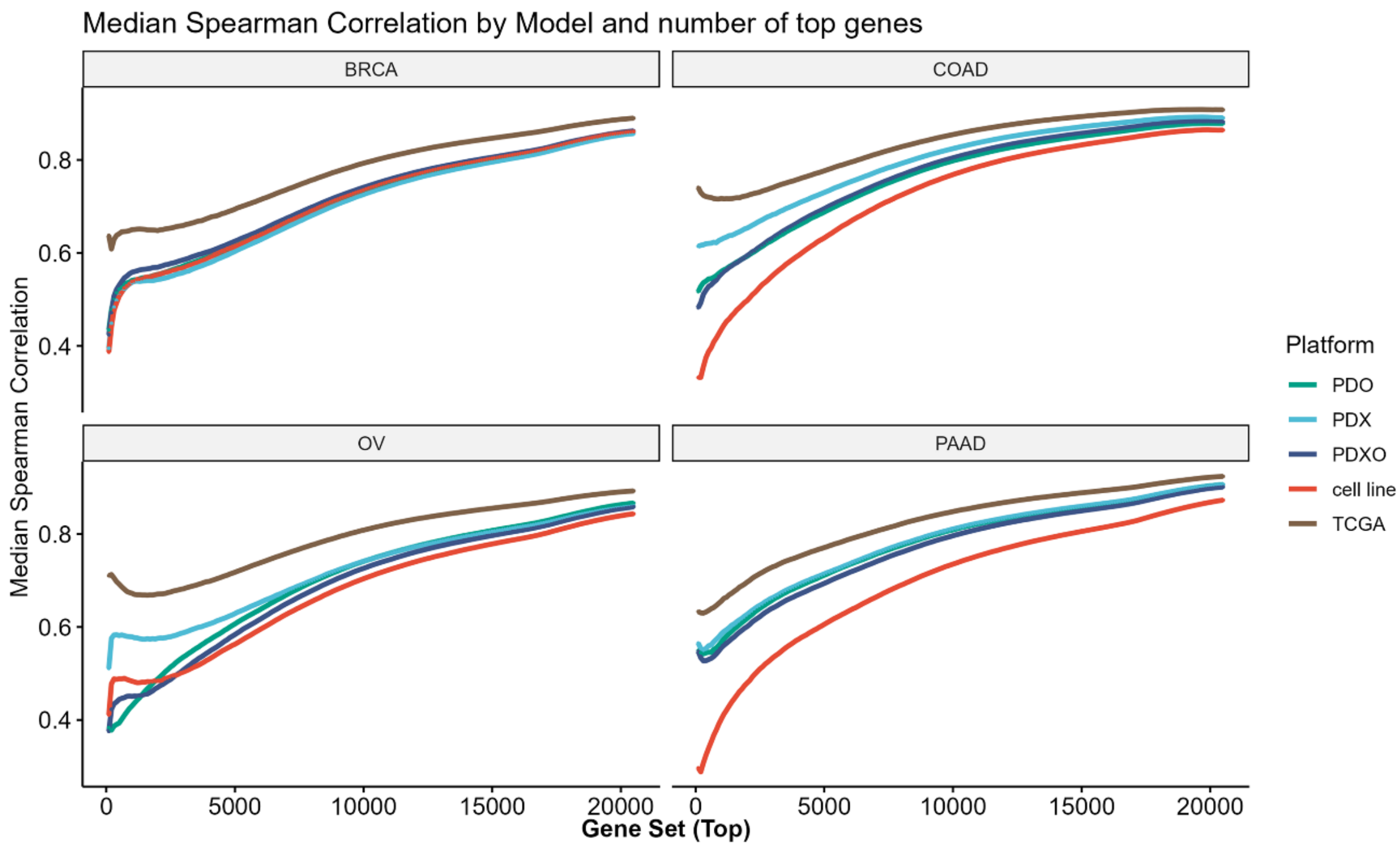

Supplementary Figure 3. Tumor type-stratified Spearman correlation analysis between preclinical models and TCGA primary tumors. Across all four tumor types, the hierarchy PDX ⩾ PDO ≈ PDXO > cell line is in general maintained, consistent with the pan-cancer analysis in **Figure 1, C-D**.

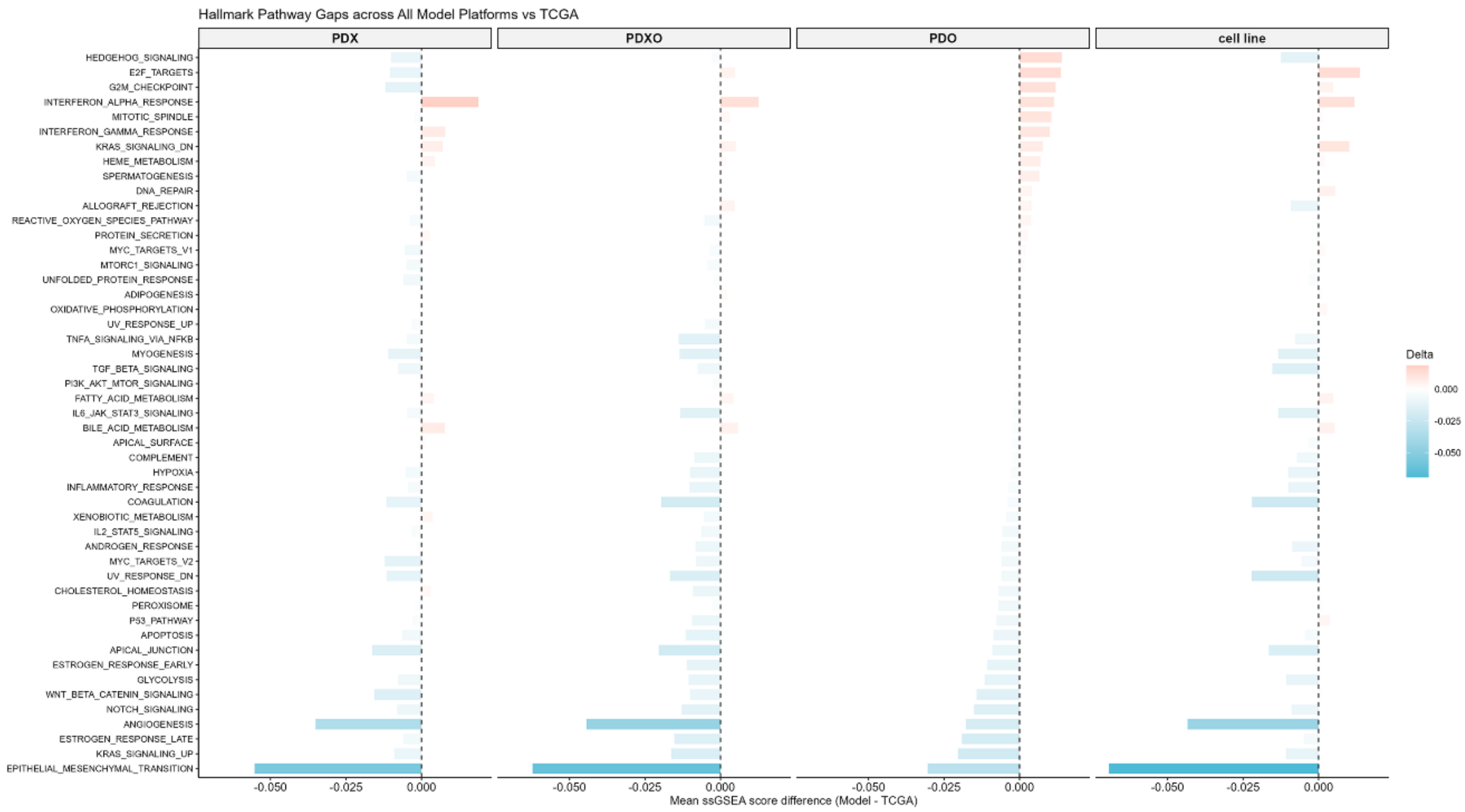

Supplementary Figure 4. Hallmark pathway enrichment gaps between preclinical cancer models and primary tumors across all model platforms. Bar plots show the mean difference in ssGSEA enrichment scores (model median − TCGA median) for each of the MSigDB Hallmark gene sets. Negative values (blue) indicate pathways underrepresented in the model relative to TCGA primary tumors; positive values (red) indicate pathways overrepresented.

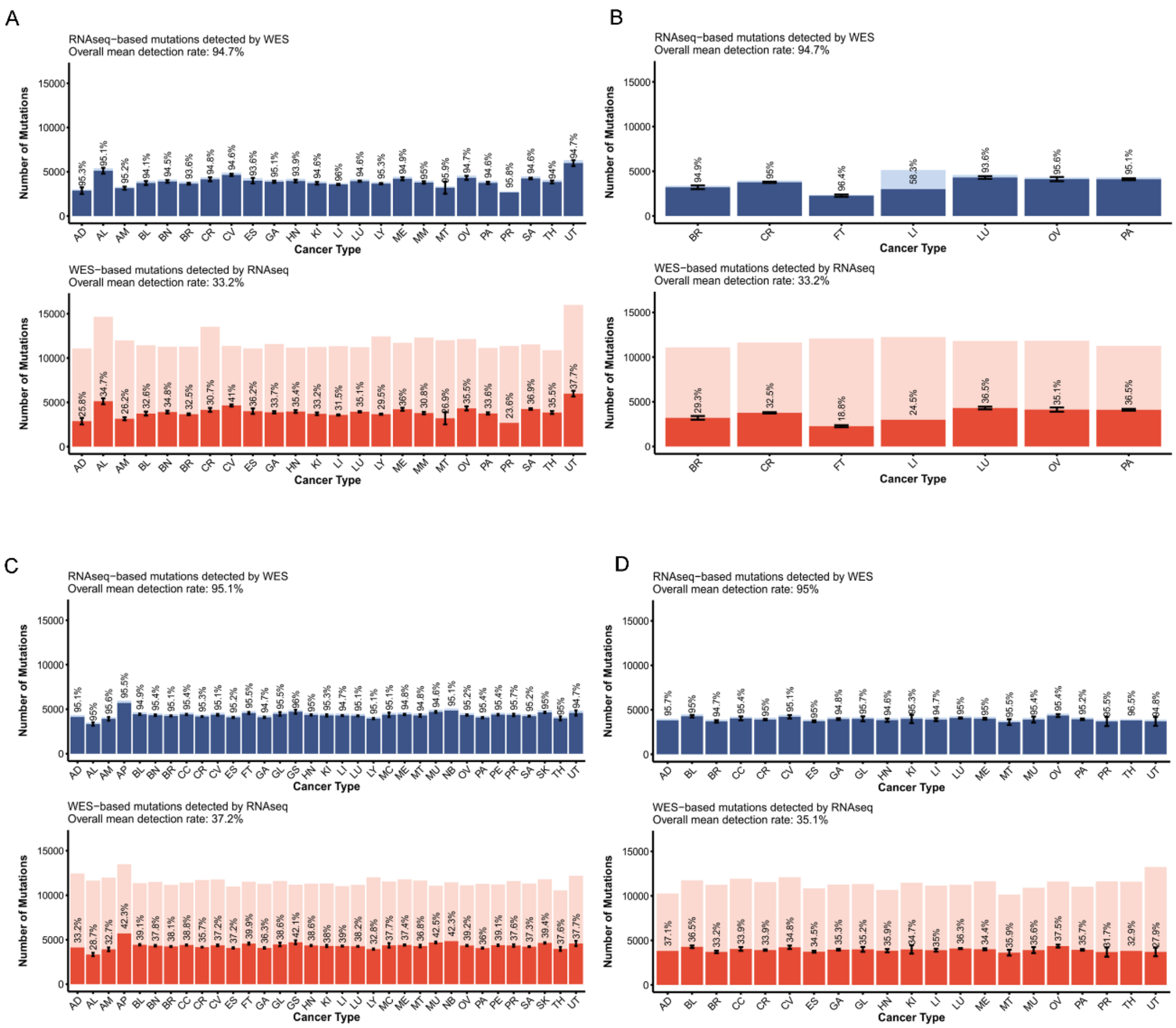

Supplementary Figure 5. WES outperforms RNA-Seq in mutation detection in preclinical tumor models. Average number of mutations identified by RNA-Seq and detection rate of WES-based mutations (upper panel) and average number of mutations identified by WES and detection rate of RNA-Seq-based mutations (lower panel) by cancer type in cell lines (**A**), PDOs (**B**), PDX (**C**) and PDXO (**D**).
